# Pirtobrutinib Monotherapy for First-Line Chronic Lymphocytic Leukemia: A Non-Anchored Indirect Comparison Using Reconstructed Patient-Level Data

**DOI:** 10.3390/hematolrep18040058

**Published:** 2026-08-14

**Authors:** Andrea Messori, Lorenzo Gasperoni, Luna Del Bono, Vera Damuzzo

**Affiliations:** 1 HTA Unit, Osservatorio Innovazione, 50134 Firenze, Italy; 2 Pharmaceutical Department, USL Toscana Centro, 59100 Prato, Italy; 3 School of Specialization in Hospital Pharmacy, University of Pisa, 56126 Pisa, Italy; 4 Department of Pharmaceutical and Pharmacological Sciences, University of Padua, 35131 Padova, Italy

**Keywords:** chlorambucil, chronic lymphocytic leukemia, ibrutinib, obinutuzumab, pirtobrutinib, progression-free survival, rituximab, venetoclax

## Abstract

**Background:** Pirtobrutinib has recently emerged as a promising first-line treatment option for chronic lymphocytic leukemia (CLL). Unlike currently established regimens, which are generally based on doublet combinations, pirtobrutinib can be administered as monotherapy. No head-to-head trials comparing pirtobrutinib with contemporary first-line combinations are currently available; hence, indirect comparative evidence may help define its potential role. **Methods:** A non-anchored indirect comparison based on reconstructed individual patient data (IPD) was conducted using published Kaplan–Meier curves from randomized controlled trials evaluating first-line treatments for CLL. Progression-free survival (PFS) was the endpoint of interest. Reconstructed IPD were generated using WebPlotDigitizer and the IPDfromKM algorithm. Then, Kaplan–Meier curves were plotted based on these patients, and the values of the restricted mean survival time (RMST) at 36 months were determined. Regarding PFS, pirtobrutinib monotherapy was compared indirectly with acalabrutinib plus obinutuzumab, venetoclax plus obinutuzumab, and venetoclax plus ibrutinib. Hazard ratios (HRs) and 95% confidence intervals (CIs) were estimated using univariate Cox models. The non-inferiority of pirtobrutinib monotherapy versus doublet regimens was assessed according to a non-inferiority margin set at HR = 1.15. Finally, the value-based prices of the new treatments were estimated based on a willingness-to-pay threshold of euro 30,000 per disease-free year gained and compared with the corresponding real prices in the Italian market. **Results:** The analysis included four randomized trials. Compared with pirtobrutinib monotherapy, HRs for PFS were 0.5544 (95%CI, 0.2696–1.1397) versus venetoclax plus obinutuzumab, 0.4583 (95%CI, 0.2066–1.0200) versus venetoclax plus ibrutinib, and 1.4453 (95%CI, 0.6684–3.1240) versus acalabrutinib plus obinutuzumab. Pirtobrutinib met the non-inferiority criterion compared with venetoclax plus obinutuzumab and venetoclax plus ibrutinib, but not with acalabrutinib plus obinutuzumab; however, these results were negatively affected by the small number of events in the pirtobrutinib arm, which generated wide CIs. In the preliminary pharmacoeconomic analysis, venetoclax plus obinutuzumab showed the most favorable profile in the comparison of value-based price vs. real price. **Conclusions:** This exploratory non-anchored analysis suggests that pirtobrutinib monotherapy may provide PFS outcomes broadly comparable to current first-line combination regimens for CLL. Given the methodological limitations inherent to indirect comparisons, prospective head-to-head studies are needed to clarify the optimal positioning of pirtobrutinib in treatment-naïve CLL.

## 1. Introduction

In the first-line treatment of chronic lymphocytic leukemia (CLL), pirtobrutinib represents a novel therapeutic option, as it can be administered as monotherapy, whereas doublet or triplet combinations are currently the standard of care (SOC). A pivotal randomized trial by Jurczak et al. [1] demonstrated that pirtobrutinib significantly improved the progression-free survival compared with bendamustine plus rituximab. Other established first-line treatment options include (i) acalabrutinib plus obinutuzumab (ELEVATE-TN trial [2,3]), (ii) venetoclax plus obinutuzumab (CLL14 trial [4]), and (iii) venetoclax plus ibrutinib (GLOW trial [5]). All of these have shown substantial improvements in PFS versus chlorambucil plus obinutuzumab. However, no head-to-head trials comparing pirtobrutinib with these doublet regimens are currently available. Pirtobrutinib has also been tested in patients with pretreated CLL (see, for example, the trial by Yi et al. [6]), but this clinical indication is outside the scope of the present report.

Indirect comparisons are often used when direct comparative evidence is lacking [7,8,9,10,11]. Such comparisons are considered to be particularly robust when treatments are linked through a common comparator (“anchored” comparisons), whereas comparisons without a shared comparator (“non-anchored” comparisons) are inherently more uncertain. The reconstruction of individual patient data (IPD) from published Kaplan–Meier curves has become a widely used method for performing indirect comparisons based on time-to-event outcomes [12,13,14].

A recent publication presented an anchored indirect comparison of first-line regimens for chronic lymphocytic leukemia (CLL) based on reconstructed IPD, including acalabrutinib plus obinutuzumab, venetoclax plus obinutuzumab, and chlorambucil plus obinutuzumab [7]. In the present analysis, this framework was extended by including an additional doublet regimen (venetoclax plus ibrutinib, GLOW trial [5]), which was not included in the previously published analysis [7], and more importantly, pirtobrutinib monotherapy [1], as additional comparators, to explore their potential position among first-line treatments, especially for pirtobrutinib monotherapy. Venetoclax plus ibrutinib was added as an “anchored” comparator, and pirtobrutinib monotherapy was added as a “non-anchored” comparator. To our knowledge, this is the first exploratory indirect comparison evaluating first-line pirtobrutinib monotherapy against contemporary fixed-duration and BTK-based combination regimens using reconstructed IPD.

Finally, our study included a preliminary pharmacoeconomic analysis using an original approach based on the above-mentioned indirect comparisons, in which the clinical results of the various pharmacological treatments were evaluated in terms of their incremental cost versus chlorambucil plus obinutuzumab. For the sake of homogeneity, all the clinical results of this pharmacoeconomic analysis are expressed as the restricted mean survival time (RMST) [15,16]. The results of this analysis estimated the value-based price (VBP) and the incremental cost-effectiveness ratio (ICER) of each treatment.

The estimation of the VBP for innovative pharmacological treatments, as well as for high-technology medical devices, is an evolving field. Over the past few years, several approaches have been proposed, ranging from complex Markov models incorporating numerous variables [17,18,19,20,21,22,23] to simplified methods [24] intended to overcome the lack of a widely accepted VBP methodology. Two major issues remain unresolved. First, the clinical evidence component is often handled through Markov modeling, although this approach is not specifically designed to analyze clinical-evidence-based datasets. Second, the economic component frequently relies on a large number of cost parameters derived from heterogeneous sources [17,18,19,20,21,22,23], which reduces the reliability of the final economic results.

The simplified method used in our pharmacoeconomic analysis is aimed at limiting the variability of the results; on the other hand, it is less frequently applicable than other methods commonly used.

## 2. Materials and Methods

### 2.1. The Method of IPD Reconstruction Based on Kaplan–Meier Curves

In our analysis, we generated reconstructed IPD from published Kaplan–Meier (KM) curves. First, we used the WebPlotDigitizer software (version 4.7; Automeris LLC, Dublin, CA, USA) to extract the X–Y coordinates; then, we used the IPDfromKM algorithm [12,13,14] to reconstruct the survival times and censoring information. Progression-free survival (PFS) was the endpoint of interest. The methodology adopted here has been previously employed in a large number of similar studies [14]. Briefly, WebPlotDigitizer converts each KM curve into 60–100 X–Y pairs that accurately represent the curve. The IPDfromKM software (www.trialdesign.org/one-page-shell.html#IPDfromKM; accessed on 2 July 2026) uses a validated reconstruction algorithm to generate a database of reconstructed individual patient data. This analysis considers the X–Y coordinates of the curve, the total number of patients, and the total number of events. The reliability of this reconstruction is supported by a large body of research. We used the online version of IPDfromKM (Version: 1.2.5.0; Last Update: 4 March 2026) (www.trialdesign.org/one-page-shell.html#IPDfromKM; accessed on 2 July 2026).

### 2.2. Design of Our Analysis

Apart from pirtobrutinib monotherapy and the GLOW trial, our analysis was based on the same trials that were evaluated in our previous report, in which two first-line treatments were assessed: venetoclax + obinutuzumab with a seven-year follow-up and acalabrutinib + obinutuzumab with a six-year follow-up. Chlorambucil + obinutuzumab was the reference treatment given to the control group. Therefore, in comparison with our previous paper, the present analysis also included pirtobrutinib monotherapy as an additional first-line treatment, according to the findings of the recent randomized controlled trial by Jurczak et al. [1], as well as the combination of venetoclax and ibrutinib, according to the GLOW trial [5]. Finally, the value-based prices of the new treatments were estimated using a willingness-to-pay threshold of €30,000 per disease-free year gained. These value-based prices were then compared with the actual prices in the Italian market, as reported on the website of the Italian Regulatory Agency (AIFA) (https://www.aifa.gov.it/). This simplified exploratory exercise was intended only to contextualize the clinical findings from a health technology assessment perspective.

### 2.3. Pharmacoeconomic Analysis

One advantage of this method is that its application is strictly limited to situations where several new treatments targeting the same disease condition have each been compared with the current standard of care (SOC). In addition, two further requirements must be met: (i) the clinical trial endpoint must be a time-to-event outcome reported via a Kaplan–Meier curve and (ii) the studies evaluating the various treatments versus SOC should have been previously published in an article based on the IPDfromKM method [12,13,14] in which minimal cross-trial heterogeneity has been found. Of course, on the one hand, these strict requirements considerably reduce the degree of applicability of the method, but on the other hand, they strongly support the quality of the final parameter estimates.

When all these conditions are satisfied, the proposed method follows three sequential steps: (i) estimating the RMST [15,16] for each treatment and for the pooled SOC control group, (ii) calculating the incremental benefit associated with each new treatment as the difference between its RMST and that of the control group, and (iii) estimating the VBP [17,18,19,20,21,22,23] of each treatment according to the following simple equation:

*VBP of the new treatment* =  (*cost of SOC*) + (*gain in years per patient*) × (*willingness-to-pay threshold*)

The willingness-to-pay threshold is set at €60,000 per life-year gained for overall survival endpoints, and at €30,000 per event-free year gained when the endpoint involves disease progression or the occurrence of a predefined adverse outcome. These valuation criteria align with the well-established Q-TWiST approach [25,26].

### 2.4. Statistical Evaluation

We estimated hazard ratios (HRs) and 95% confidence intervals (CIs) using a univariate Cox model in all of our time-to-event analyses. Furthermore, the RMST values at 36 months were determined; this truncation time was the longest follow-up reached in all the included trials. Statistical analyses and graphical representations were performed using R (version 4.5.1; R Foundation for Statistical Computing, Vienna, Austria).

As the pirtobrutinib trial used bendamustine plus rituximab as the comparator and the other trials used chlorambucil plus obinutuzumab, pirtobrutinib monotherapy could only be included as a non-anchored comparator. The non-inferiority of pirtobrutinib monotherapy versus doublet regimens was assessed by setting the non-inferiority margin at HR = 1.15. This non-inferiority margin [27] was selected as an exploratory threshold intended to represent preservation of most of the expected treatment benefit while acknowledging the uncertainties intrinsic to reconstructed-IPD non-anchored comparisons. Furthermore, the assumption of risk proportionality, which is the basis of the Cox model, was assessed by performing the Schoenfeld test [28], which determines whether this assumption is violated.

## 3. Results

Table 1 shows the main characteristics of the four randomized controlled trials (RCTs) included in our analysis.

**Figure 1:**
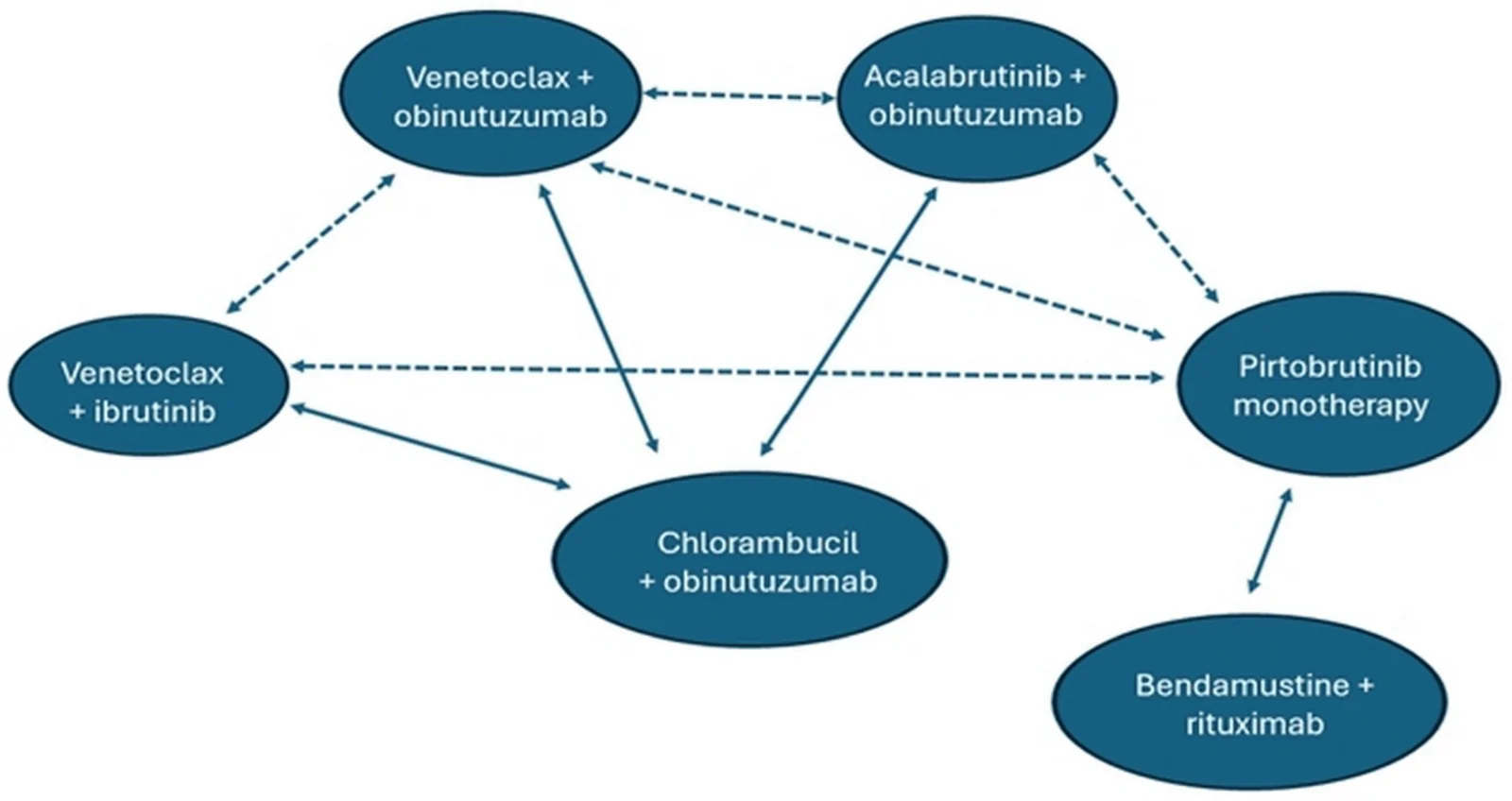
Geometry of our network survival meta-analysis: direct comparisons based on a “real” RCT are represented by a solid arrow, while indirect comparisons are represented by a dashed arrow. All indirect comparisons are anchored with the exception of the three indirect comparisons involving pirtobrutinib monotherapy, which are non-anchored. The endpoint is progression-free survival. Abbreviations: RCT, randomized controlled trial.

**Figure 2:**
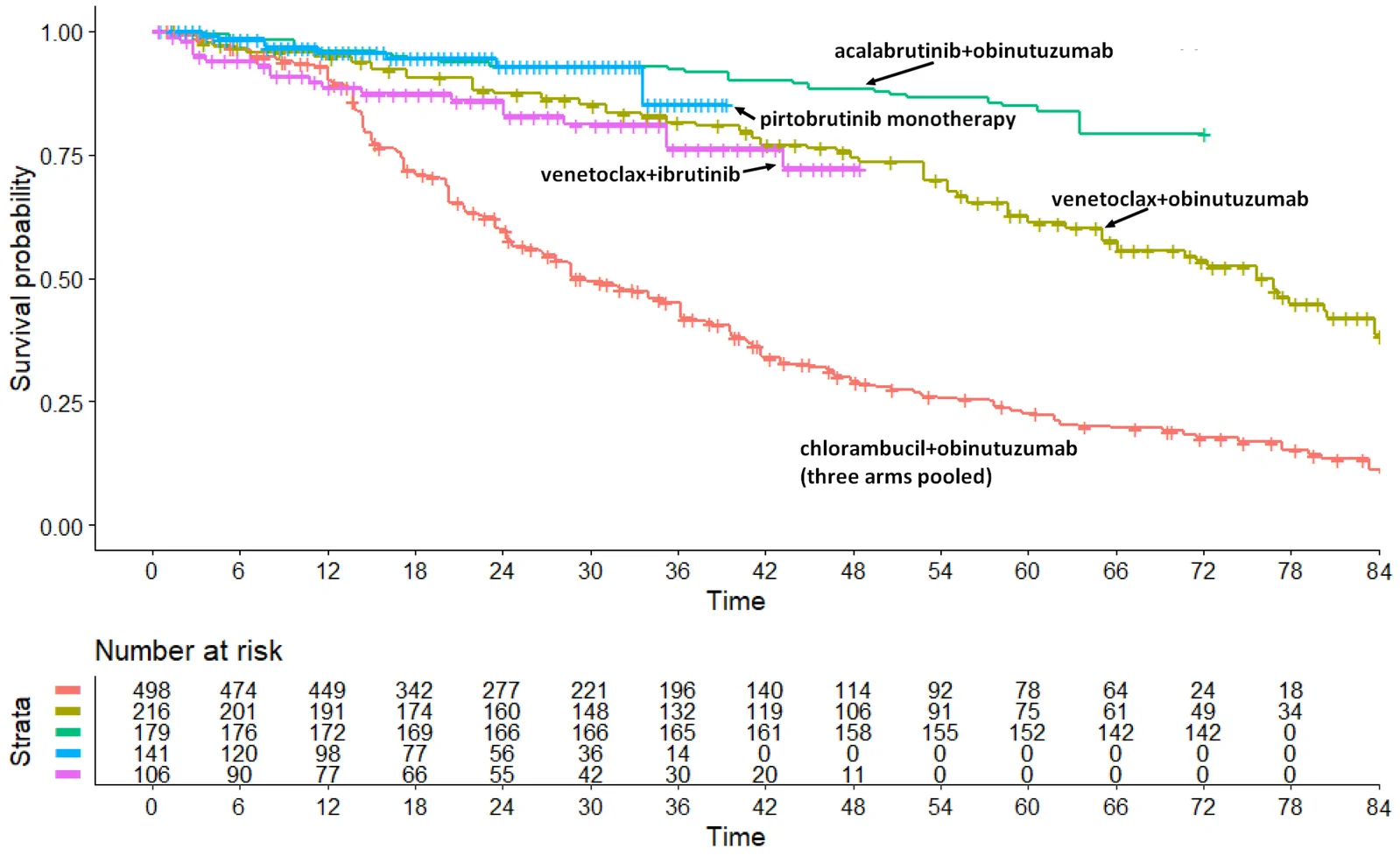
Results of the main analysis that includes three regimens anchored with one another and pirtobrutinib monotherapy as a non-anchored comparator. Time-to-event endpoint: progression-free survival. Abbreviations: PFS, progression-free survival. Time in months.

Figure 1 illustrates the geometry of our network survival meta-analysis. Figure 2 shows the reconstructed Kaplan–Meier (KM) curves for progression-free survival (PFS) across all these treatments (i.e., pirtobrutinib monotherapy, venetoclax plus obinutuzumab, venetoclax plus ibrutinib, acalabrutinib plus obinutuzumab, and chlorambucil plus obinutuzumab).

For this endpoint, the hazard ratio (HR) for pirtobrutinib monotherapy versus venetoclax plus obinutuzumab was 0.5544 (95% confidence interval (CI), 0.2696 to 1.1397), while the HR for pirtobrutinib monotherapy versus venetoclax plus ibrutinib was 0.4583 (95% CI, 0.2066 to 1.02). To evaluate the quality of the IPD reconstruction in the pirtobrutinib trial, the HR in Jurczak’s original trial in comparison with bendamustine–rituximab was 0.199 (95% CI, 0.107 to 0.367) [1], while that recomputed from reconstructed patients using bendamustine–rituximab as a comparator was 0.1713 (95%CI, 0.08354 to 0.3512). Table 2 shows the same comparison for the three trials using chlorambucil + obinutuzumab for the control groups. These results confirm the reliability of the IPDfromKM method.

Regarding non-inferiority, the non-inferiority criterion was met in the comparison of pirtobrutinib monotherapy with venetoclax plus obinutuzumab and venetoclax plus ibrutinib. By contrast, the HR for pirtobrutinib monotherapy versus acalabrutinib plus obinutuzumab was 1.4453 (95% CI, 0.6684 to 3.124), which clearly did not satisfy the non-inferiority criterion. Visual inspection suggested no major violations of the proportional hazard assumption (see Figure S1 in the Supplementary Materials). By contrast, the degree of cross-trial heterogeneity between the four control arms of the included trials was statistically significant (see Figure S2 in the Supplementary Materials); similarly, in the comparison of the three arms given chlorambucil + obinutuzumab, this heterogeneity remained statistically significant, likely because the control arm of the CLL14 trial performed better than the controls of the ELEVATE-TN and GLOW trials.

Pirtobrutinib showed no evidence of clinically relevant inferiority according to the prespecified HR threshold when compared with venetoclax plus obinutuzumab and venetoclax plus ibrutinib, but not with acalabrutinib plus obinutuzumab. However, it should be noted that the non-anchored connection of pirtobrutinib to the network geometry reduces the validity of these comparisons.

Finally, our preliminary pharmacoeconomic analysis (Table 3) showed that venetoclax plus obinutuzumab had the most favorable profile when VBPs were compared with real prices.

## 4. Discussion

The results from the indirect comparisons should be interpreted with caution, especially when the comparison is unanchored and relies on reconstructed individual patient data (IPD) rather than on the original databases of patients enrolled in clinical trials. Using this approach, differences in eligibility criteria, baseline prognostic factors, and comparator regimens across trials may introduce residual confounding that cannot be adjusted for when the analysis is based on reconstructed IPD. Nevertheless, this type of research can provide meaningful insights. In the present analysis, the preliminary evidence suggests that pirtobrutinib monotherapy may achieve PFS outcomes broadly comparable with those of established doublet regimens.

However, in our study, pirtobrutinib was compared indirectly across trials using different control arms (bendamustine + rituximab in one trial and chlorambucil plus obinutuzumab in the other three), creating a substantial risk of confounding and limiting the interpretability of the findings. First, PFS is a surrogate endpoint whose relationship with overall survival may vary across different treatment regimens. In addition, there was considerable heterogeneity between the control arms of the four included trials, as well as between the three trials in which chlorambucil plus obinutuzumab was used as the control regimen.

Despite these potential sources of confounding, our analysis supports, from a statistical perspective, the hypothesis that pirtobrutinib monotherapy may be non-inferior to venetoclax plus obinutuzumab and venetoclax plus ibrutinib. In contrast, pirtobrutinib monotherapy showed a numerically less favorable PFS pattern than acalabrutinib plus obinutuzumab.

Regarding the non-inferiority margin set at HR = 1.15, although its value is inherently arbitrary, as in most non-inferiority analyses, when overall survival or PFS is used as an endpoint in oncology studies, an HR of 1.15 is generally considered a conservative non-inferiority margin [16,29].

Overall, our findings are consistent with a recent editorial published in the Journal of Clinical Oncology, which highlighted the potential role of pirtobrutinib monotherapy in the treatment of previously untreated patients with CLL [30]. If future comparative studies confirm these findings, pirtobrutinib monotherapy could represent a simpler therapeutic approach than combination regimens.

As with all indirect comparisons, this analysis has important limitations. First, when reconstructed patient-level data are used, the Cox model can only be fitted as a univariable analysis, whereas multivariable Cox regression can be only performed when original IPD are available, including patient-level information on the main covariates. However, the IPDfromKM method offers the advantages of well-documented reproducibility and a straightforward reconstruction process.

Regarding our preliminary pharmacoeconomic analysis in the Italian setting, the prices reported on the AIFA website are ex-factory prices and do not include discounts resulting from national price negotiations, the details of which are confidential. Similarly, the willingness-to-pay threshold of €30,000 per disease-free year gained has not been formally adopted by AIFA. Therefore, the data presented in Table 3 should be regarded as hypothesis-generating rather than as fully validated results. On the other hand, VBPs provide a more comprehensive interpretation of the clinical findings. Furthermore, their validity increases when the geometry of cross-treatment clinical comparisons is relatively simple, as in the present analysis.

Finally, the results of our indirect comparisons align well with the European Hematology Association (EHA) guidelines on the management of chronic lymphocytic leukemia and Richter transformation, which were published recently [31]. Regarding treatment options, the main updates reported in these guidelines include the time-limited combination therapy of acalabrutinib and venetoclax  ±  obinutuzumab, recently approved by the European Medicines Agency (EMA) as first-line treatment, and continuous pirtobrutinib for patients exposed to covalent Bruton’s tyrosine kinase inhibitors in the relapsed/refractory setting. Regarding the use of pirtobrutinib monotherapy in untreated patients, the guidelines mention the RCT published by Jurczak et al. [1] but underscore that this treatment was not yet approved in Europe at that time. However, on 26 June 2026, the CHMP of the EMA adopted a positive opinion recommending a change to the terms of the marketing authorization for pirtobrutinib to include first-line treatment for CLL [32].

## 5. Conclusions

Following the publication of the BRUIN CLL-313 trial [1], several reports have highlighted the potential role of pirtobrutinib monotherapy in the treatment of previously untreated patients with CLL. If these findings are confirmed in prospective comparative studies, pirtobrutinib could become an attractive first-line treatment option because of its simpler administration compared with combination regimens. However, further research is needed to define its optimal place within the therapeutic landscape.

## Figures and Tables

**Table 1:** Main characteristics of the included trials.

Trial	First Author and Reference	Main Inclusion Criteria	Comparison §	Follow-Up Duration * (mos)	Treatment Group		Control Group	
					Events (N)	Patients (N)	Events (N)	Patients (N)
BRUIN CLL-313	Jurczak [1]	Patients with previously untreated chronic lymphocytic leukemia without del (17p).	P monotherapy vs. R + B	38	13	141	48	141
ELEVATE- TN	Sharam [2,3]	Patients with untreated chronic lymphocytic leukemia, aged 65 years or older, or older than 18 years and younger than 65 years with creatinine clearance of 30–69 mL/min (calculated by use of the Cockcroft–Gault equation) or Cumulative Illness Rating Scale for Geriatrics score greater than 6.	A + O vs. C + O vs. A monotherapy §§	72	37	179	146	177
CLL14	Al-Sawaf [4]	Patients who had previously untreated CD20+ chronic lymphocytic leukemia who had been diagnosed in accordance with the criteria of the International Workshop on CLL and had been determined by the treating clinician and confirmed during the central screening process to require therapy (Binet stage C [low hemoglobin or platelet count from bone marrow infiltration of CLL cells] or symptomatic disease).	V + O vs. C + O	78	82	216	152	216
GLOW	Niemann [5]	Patients aged 65 years and older or 18–64 years with previously untreated chronic lymphocytic leukemia and a cumulative illness rating scale score of more than 6 or creatinine clearance less than 70 mL/min, or both, and an Eastern Cooperative Oncology Group performance status of 2 or less.	V + I vs. C + O	48	29	106	78	105

* Last-time point in the Kaplan–Meier graph. § In the four trials, progression-free survival was the endpoint for our analysis. §§ This arm was not evaluated in our analysis. Abbreviations: PFS, progression-free survival; mos, months; I, ibrutinib; O, obinutuzumab; P, pirtobrutinib; R, rituximab; B, bendamustine; C, chlorambucil; V, venetoclax.

**Table 2:** Comparison between original HR values and those estimated from reconstructed patients.

Trial	First Author and Reference	Treatments Given to the Experimental Arm and the Control Arm	HR of Treatment Arm vs. Controls Reported in the Original Trial	HR of Treatment Arm vs. Controls Computed from Reconstructed Patients
BRUIN CLL-313	Jurczak [1]	P monotherapy vs. R + B	0.199 95%CI, 0.107 to 0.367	0.1713 95%CI, 0.08354 to 0.3512
ELEVATE- TN	Sharam [2,3]	A + O vs. C + O	0·10 95%CI, 0.06 to 0.17	0.126 95%CI, 0.08721 to 0.1821
CLL14	Al-Sawaf [4]	V + O vs. C + O	0.40 95%CI, 0.31 to 0.52	0.3937 95%CI, 0.3004 to 0.516
GLOW	Niemann [5]	V + I vs. C + O	0.214 95%CI, 0.138 to 0·334	0.2386 95%CI, 0.1437 to 0.396

Abbreviations: CI, confidence interval; mos, months; I, ibrutinib; O, obinutuzumab; P, pirtobrutinib; R, rituximab; B, bendamustine; C, chlorambucil; V, venetoclax.

**Table 3:** Values of RMST (from 0 to 36 months) estimated for the four new treatments and the SOC and consequent estimates of value-based price.

Trial §	First Author and Reference	New Treatment	RMST * (mos)	Gain in Comparison with SOC (mos)	Real Cost of SOC † (Euro/Patient)	Estimated Value-Based Cost of the New Treatment (Euro/Patient) †	Real Cost of the New Treatment † (Euro/Patient)
BRUIN CLL-313	Jurczak [1]	P monotherapy	34.158 (95%CI, 32.950 to 35.366)	7.697 §§	24,000	254,910	354,000
ELEVATE-TN	Sharam [2,3]	A + O	34.325 (95%CI, 33.414 to 35.236)	7.864		259,920	245,000
CLL14	Al-Sawaf [4]	V + O	32.814 (95%CI, 31.706 to 33.923)	6.353		214,590	96,000
GLOW	Nieman [5]	V + I	31.530 (95%CI, 29.494 to 33.565)	5.069		176,070	163,000
SOC (data pooled from three trials)	[3,4,5]	C + O	26.461 (95%CI, 25.531 to 27.392)	-		-	

§ In the four trials, progression-free survival was the endpoint for our analysis. * With respect to the endpoint of PFS. † These three parameters are based on a treatment duration equal to RMST. §§ In this trial, SOC was bendamustine + rituximab and not chlorambucil + obinutuzumab. Abbreviations: PFS, progression-free survival; CI, confidence interval; mos, months; I, ibrutinib; O, obinutuzumab; P, pirtobrutinib; R, rituximab; B, bendamustine; C, chlorambucil; V, venetoclax.

## Data Availability

For each of the treatments under comparison, the database of reconstructed patients is available from the authors upon request.

## Supplementary Materials

The following supporting information can be downloaded at https://www.mdpi.com/article/10.3390/hematolrep18040058/s1, Figure S1. Schoenfeld’s residuals graph. The global analysis of all data is represented by the solid line in blue while the five treatment groups can be identified as follows: in dark purple, chlorambucil + obinutuzumab, all control groups pooled; in orange, venetoclax + obinutuzumab, Al-Sawaf’s trial [4]; in light violet, acalabrutinib + obinutuzumab, Sharam’s trial [2,3]; in grey, pirtobrutinib monotherapy, Jurczak’s trial [1]; in green, venetoclax + ibrutinib, Niemann’s trial [5]. By visual inspection of this graph, each of the five treatment groups shows a horizontal trend confirming that the risk proportionality is not violated. By contrast, the global analysis of all data (solid line in blue) shows, as expected, deviations from a horizontal trend (with *p* < 0.05), due to the difference in outcomes across the four experimental treatment groups compared with the pooled control group. Figure S2. Kaplan-Meier curves of the 4 control arms of included trials. The degree of cross-trial heterogeneity among the 4 control arms is statistically significant (*p* < 0.01), and remains significant at the same level (*p* < 0.01) even if only the three arms given chlorambucil+ obinutuzumab are kept in the analysis. Time in months.

## References

[1] Jurczak W., Kwiatek M., Czyz J., Roberto de Mattos E., Eom K.S., Egle A., Wrobel T. (2026). BRUIN CLL- 313: Randomized Phase III Trial of Pirtobrutinib Versus Bendamustine Plus Rituximab in Untreated Patients with Chronic Lymphocytic Leukemia/Small Lymphocytic Lymphoma. J. Clin. Oncol..

[2] Sharman J.P., Egyed M., Jurczak W., Skarbnik A., Pagel J.M., Flinn I.W., Byrd J.C. (2020). Acalabrutinib with or without obinutuzumab versus chlorambucil and obinutuzmab for treatment-naive chronic lymphocytic leukemia (ELEVATE TN): A randomised, controlled, phase 3 trial. Lancet.

[3] Sharman J. (2024). Six-year update of the ELEVATE-TN study on acalabrutinib. Clin. Adv. Hematol. Oncol..

[4] Al-Sawaf O., Robrecht S., Zhang C., Olivieri S., Chang Y.M., Fink A.M., Fischer K. (2024). Venetoclax-obinutuzumab for previously untreated chronic lymphocytic leukemia: 6-year results of the randomized phase 3 CLL14 study. Blood.

[5] Niemann C.U., Munir T., Moreno C., Owen C., Follows G.A., Benjamini O., Kater A.P. (2023). Fixed-duration ibrutinib-venetoclax versus chlorambucilobinutuzumab in previously untreated chronic lymphocytic leukemia (GLOW): 4-year follow-up from a multicentre, open-label, randomised, phase 3 trial. Lancet Oncol..

[6] Yi S., Cao J., Feng R., Zhou K., Qiu L., Li F., Qiu L. (2026). A comprehensive analysis of pirtobrutinib in Chinese patients with chronic lymphocytic leukemia/small lymphocytic lymphoma (CLL/SLL): Results from the phase 3 study BRUIN CLL-321. Br. J. Haematol..

[7] Messori A. (2026). Updating efficacy of first-line treatments for chronic lymphocytic leukemia: Indirect comparisons based on recent data favor the combination of acalabrutinib plus obinutuzumab (Letter). Eur. J. Hematol..

[8] Veroniki A.A., Straus S.E., Soobiah C., Elliott M.J., Tricco A.C. (2016). A scoping review of indirect comparison methods and applications using individual patient data. BMC Med. Res. Methodol..

[9] Farinasso C.M., Ferreira V.L., Medeiros F.C., da Rocha A.P., Parreira P.D.C.S., Oliveira L.A., de Oliveira H.A. (2025). Matching-Adjusted Indirect Comparison Studies in Oncology a Scoping Review Focused on Reporting Quality. Value Health Reg. Issues.

[10] Cassidy O., Harte M., Trela-Larsen L., Walsh C., White A., McCullagh L., Leahy J. (2023). A Comparison of Relative-Efficacy Estimate(s) Derived from Both Matching-Adjusted Indirect Comparisons and Standard Anchored Indirect Treatment Comparisons: A Review of Matching-Adjusted Indirect Comparisons. Value Health.

[11] Margolin K.A. (2024). Using Indirect Comparisons of Prospective, Randomized Trials to Make Therapeutic Decisions in Melanoma: Cross-Trial Comparisons as Surrogates for Proper Head-To-Head Studies?. J. Clin. Oncol..

[12] Guyot P., Ades A.E., Ouwens M.J., Welton N.J. (2012). Enhanced secondary analysis of survival data: Reconstructing the data from published Kaplan-Meier survival curves. BMC Med. Res. Methodol..

[13] Liu N., Zhou Y., Lee J.J. (2021). IPDfromKM: Reconstruct individual patient data from published Kaplan-Meier survival curves. BMC Med. Res. Methodol..

[14] Messori A. Reconstruction of Individual-Patient Data from the Analysis of Kaplan-Meier Curves: This Method Is Widely Used in Oncology and Cardiology-List of 173 Studies in Oncology and 106 in Cardiology (Preprint). Open Science Framework. Published 11 March 2026. https://osf.io/srtca/overview.

[15] Royston P., Parmar M.K. (2013). Restricted mean survival time: An alternative to the hazard ratio for the design and analysis of randomized trials with a time-to-event outcome. BMC Med. Res. Methodol..

[16] Bartoli L., Ferracane E., Trippoli S., Messori A. (2021). First-line treatments for chronic lymphocytic leukemia: Analysis of 7 trials based on the restricted mean survival time. Int. J. Clin. Pharmacol. Ther..

[17] Briggs A., Claxton K., Sculpher M. (2006). Decision Modelling for Health Economic Evaluation.

[18] Woods B.S., Sideris E., Palmer S.J., Latimer N., Soares M.F.O. (2017). NICE DSU Technical Support Document 19: Partitioned Survival Analysis for Decision Modelling in Health Care: A Critical Review.

[19] Gonçalves E. (2022). Value-based pricing for advanced therapy medicinal products: Emerging affordability solutions. Eur. J. Health Econ..

[20] Feenstra T., Corro-Ramos I., Hamerlijnck D., van Voorn G., Ghabri S. (2022). Four aspects affecting health economic decision models and their validation. Pharmacoeconomics.

[21] Jommi C., Armeni P., Costa F., Bertolani A., Otto M. (2020). Implementation of value-based pricing for medicines. Clin. Ther..

[22] Tremblay G., Poirier A., Monfort L. (2024). Value-based pricing: A potential solution to difficult pricing discussions and payers’ negotiations. J. Med. Econ..

[23] Hyeraci G., Trippoli S., Rivano M., Messori A. (2023). Estimation of value-based price for 48 high-technology medical devices. Cureus.

[24] Messori A., Tognarelli B., Monzillo J., Trippoli S. (2025). Simplified Algorithm for Determining Value-Based Pricing of High-Tech Medical Devices in the Tuscany Region of Italy. Cureus.

[25] Goldhirsch A., Gelber R.D., Simes R.J., Glasziou P., Coates A.S. (1989). Costs and benefits of adjuvant therapy in breast cancer: A quality-adjusted survival analysis. J. Clin. Oncol..

[26] Gelber R.D. (1993). Evaluation of effectiveness: Q-TWiST. Cancer Treat. Rev..

[27] Piragine E., Trippoli S., Veneziano S., Messori A., Calderone V. (2025). The Reconstructed Individual Patient Data from Kaplan-Meier (IPDfromKM) Method. for Non-Inferiority Analyses: A new potential. application. Methods Protoc..

[28] Schoenfeld D. (1982). Partial Residuals for the Proportional Hazards Regression Model. Biometrika.

[29] Eichhorst B., Fink A.M., Bahlo J., Busch R., Kovacs G., Maurer C., Hallek M. (2016). First-line chemoimmunotherapy with bendamustine and rituximab versus fludarabine, cyclophosphamide, and rituximab in patients with advanced chronic lymphocytic leukaemia (CLL10): An international, open-label, randomised, phase 3, non-inferiority trial. Lancet Oncol..

[30] Braunstein M.J., Williams M.E. (2026). Bridging the Gap: Pirtobrutinib for Treatment-Naïve Chronic Lymphatic Leukemia. J. Clin. Oncol..

[31] Eichhorst B., Ghia P., Bosch F., Clifford R., Gregor M., Guieze R., York N. (2026). EHA Guidelines on management of chronic lymphocytic leukemia and Richter transformation. HemaSphere.

[32] European Medicines Agency Website Jaypirca (Pirtobrutinib): Opinion on Variation to Marketing Authorization. https://www.ema.europa.eu/en/medicines/human/variation/jaypirca.

