# Supplementary material for: Pirtobrutinib Monotherapy for First-Line Chronic Lymphocytic Leukemia: A Non-Anchored Indirect Comparison Using Reconstructed Patient-Level Data
Source: Hematol Rep. 2026 Aug 14;18(4):58. doi: 10.3390/hematolrep18040058 (PMC13512206; doi:10.3390/hematolrep18040058)

SUPPLEMENTARY MATERIAL

Figure S1. Schoenfeld's residuals graph. The global analysis of all data is represented by the solid line in blue while the five treatment groups can be identified as follows: in dark purple, chlorambucil+obinutuzumab, all control groups pooled; in orange, venetoclax+obinutuzumab, Al-Sawaf's trial [4]; in light violet, acalabrutinib+obinutuzumab, Sharam's trial [2,3]; in grey, pirtobrutinib monotherapy, Jurczak's trial [1]; in green, venetoclax+ibrutinib, Niemann's trial [5]. By visual inspection of this graph, each of the five treatment groups shows a horizontal trend confirming that the risk proportionality is not violated. By contrast, the global analysis of all data (solid line in blue) shows, as expected, deviations from a horizontal trend (with $p<0.05$), due to the difference in outcomes across the four experimental treatment groups compared with the pooled control group.

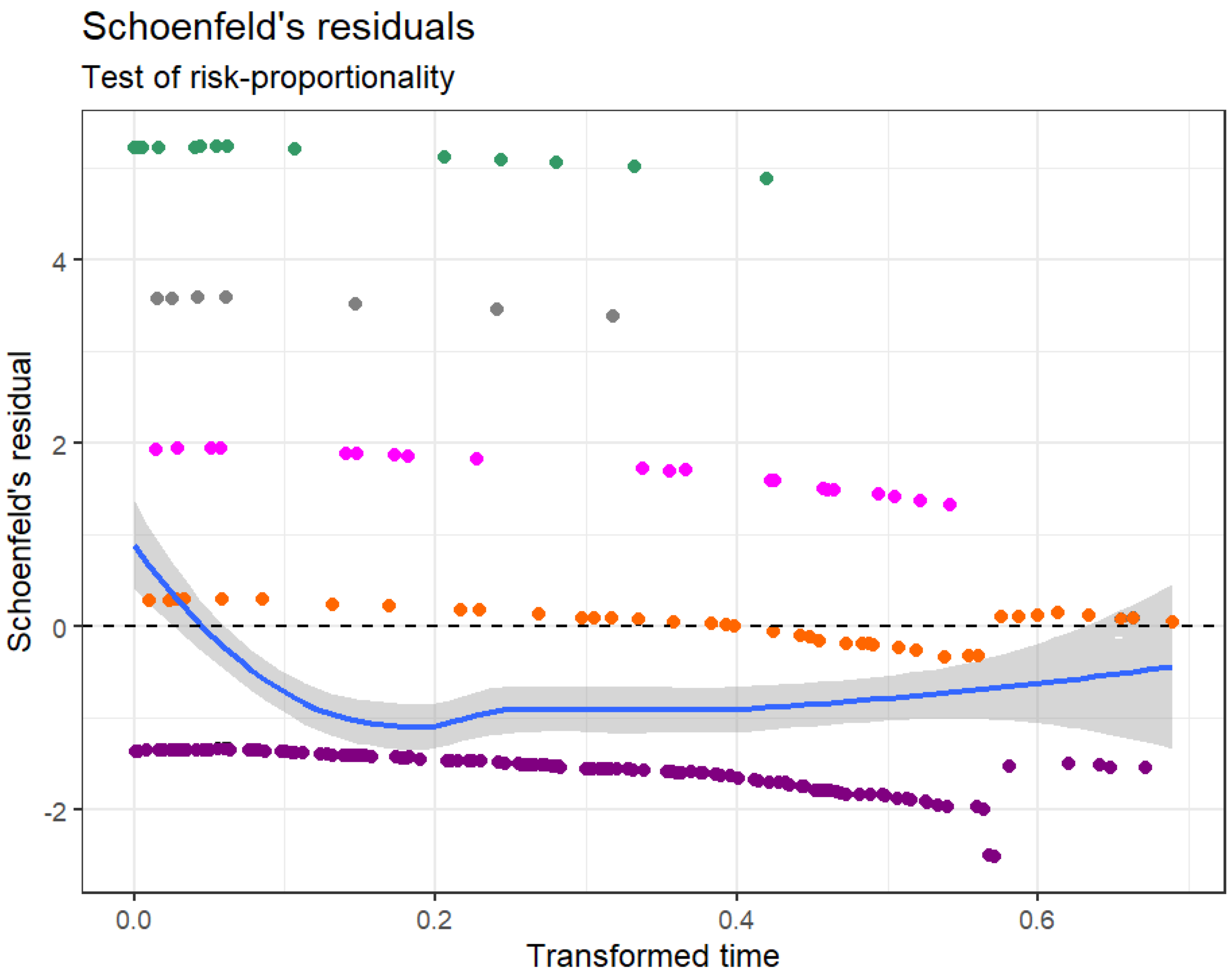

Figure S2. Kaplan-Meier curves of the 4 control arms of included trials. The degree of cross-trial heterogeneity among the 4 control arms is statistically significant (p<0.01), and remains significant at the same level (p<0.01) even if only the three arms given chlorambucil+ obinutuzumab are kept in the analysis. Time in months.

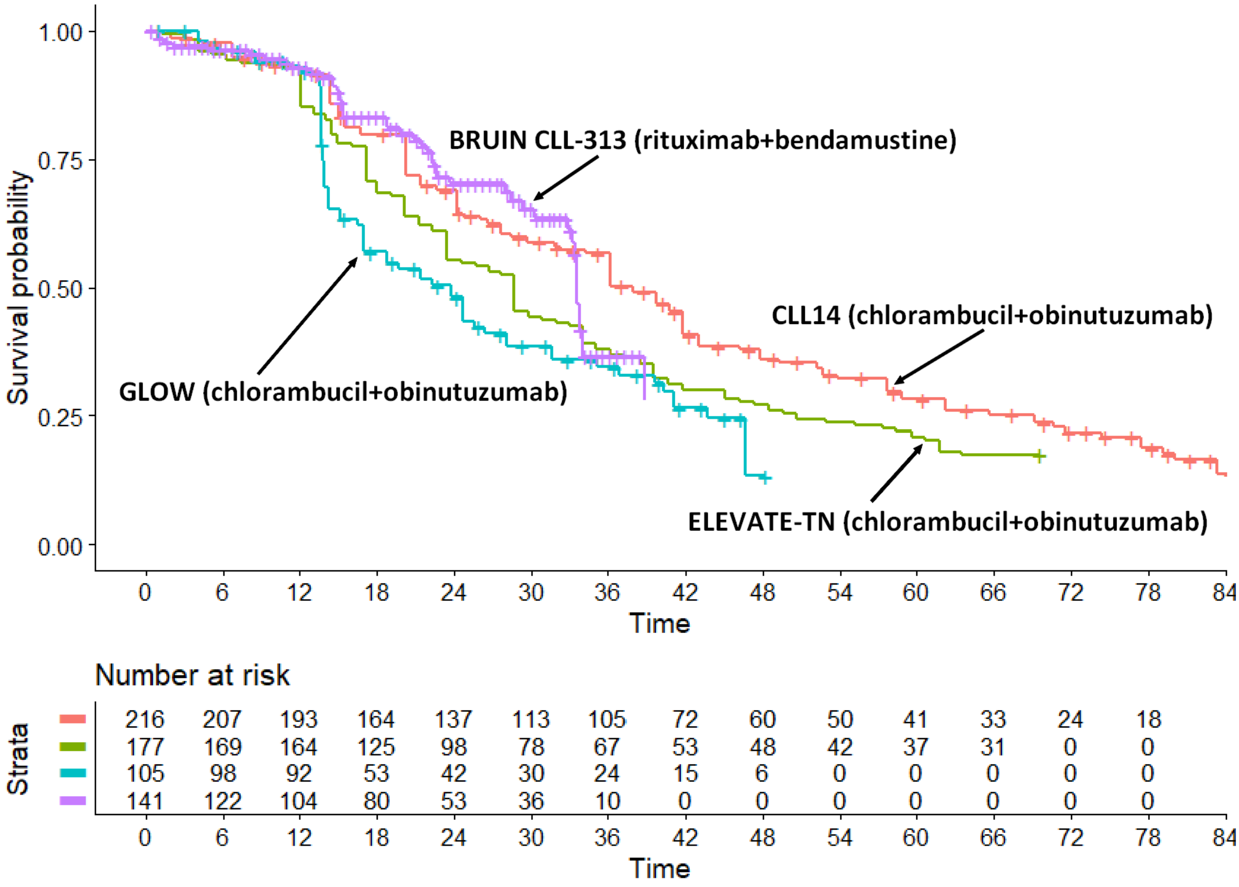

Supplement: Supplementary file 1 [file hematolrep-18-00058-s001.zip › hematolrep-4344546-supplementary.pdf]
